# Application of Turkevich Method for Gold Nanoparticles Synthesis to Fabrication of SiO_2_@Au and TiO_2_@Au Core-Shell Nanostructures

**DOI:** 10.3390/ma8062849

**Published:** 2015-05-26

**Authors:** Paulina Dobrowolska, Aleksandra Krajewska, Magdalena Gajda-Rączka, Bartosz Bartosewicz, Piotr Nyga, Bartłomiej J. Jankiewicz

**Affiliations:** Institute of Optoelectronics, Military University of Technology, Warsaw 00-908, Poland; E-Mails: paulina.doborowolska@student.wat.edu.pl (P.D.); aleksandra.krajewska@wat.edu.pl (A.K.); magdalena.gajda-raczka@wat.edu.pl (M.G.R.); bartosz.bartosewicz@wat.edu.pl (B.B.); piotr.nyga@wat.edu.pl (P.N.)

**Keywords:** SiO_2_@Au, TiO_2_@Au, core-shell nanostructures, Au nanoparticles, Turkevich Method

## Abstract

The Turkevich synthesis method of Au nanoparticles (AuNPs) was adopted for direct fabrication of SiO_2_@Au and TiO_2_@Au core-shell nanostructures. In this method, chloroauric acid was reduced with trisodium citrate in the presence of amine-functionalized silica or titania submicroparticles. Core-shells obtained in this way were compared to structures fabricated by mixing of Turkevich AuNPs with amine-functionalized silica or titania submicroparticles. It was found that by modification of reaction conditions of the first method, such as temperature and concentration of reagents, control over gold coverage on silicon dioxide particles has been achieved. Described method under certain conditions allows fabrication of semicontinuous gold films on the surface of silicon dioxide particles. To the best of our knowledge, this is the first report describing use of Turkevich method to direct fabrication of TiO_2_@Au core-shell nanostructures.

## 1. Introduction

In recent years, nanotechnology is one of the fastest growing fields in science and engineering. One of the results of extremely rapid progress in this field is the discovery of the large number of functional materials in which at least one dimension is in the range of 1 to 100 nm. Some of the most prominent examples of these materials are noble metal nanostructures, such as nanoparticles (NPs) [1], nanowires [2,3], nanorods [3,4,5], nanotriangles [6], nanostars [7] and many others. Various properties and thus also possible applications of noble metal nanostructures, are dependent on their composition, shape and size [8,9,10]. The noble metals were also used for fabrication of more complex hybrid nanostructures, where they are combined with other materials. This often results in new functionalities as compared to nanostructures made from noble metal only. The core-shell nanostructures consisting of oxide core and noble metal shell is an interesting example of such hybrid nanostructures.

The core-shell nanostructures have received increasing attention since Halas and co-workers [11] reported studies on the synthesis of SiO_2_@Au nanoparticles. In this work, a continuous gold nanoshell was grown on the surface of amine-functionalized silica in a two-step process. The amine-functionalized silica particles were decorated with very small Au nanoparticles (1–2 nm in diameter) in the first step. In the next step, the growth of the shell on gold-decorated silica particles was realized by a subsequent reduction of an aged mixture of chloroauric acid and potassium carbonate by a solution of sodium borohydride. The Au nanoparticles on silica surface served as a nucleation sites for the reduction. One of the most important characteristics of the core-shell nanostructures is that their optical properties, localized surface plasmon resonance (LSPR) band positions, can be tuned by their geometry, core radius/shell thickness ratio. The possibility of core-shell nanostructures optical properties tuning has opened the door to many applications such as cancer therapy [12], surface enhanced spectroscopies [13,14] or steam generation for solar autoclave [15].

Following work of Halas [11], several approaches to fabrication of core-shell nanostructures based on the silica core and gold shell have been reported [16,17,18,19,20,21,22,23,24,25,26,27,28,29,30,31,32,33]. The most commonly used approach to synthesis of core-shell nanostructures based on silica and gold involves a two-step process, deposition of gold seeds on functionalized silica surface, followed by gold shell growth. Research studies based on this approach were mainly focused on the investigations of the influence of silica surface functionalization and reducing agent used for shell growth on the formation of gold shell.

The silica surface functionalization is crucial for deposition of small gold nanoparticles, which serve as seeds for shell growth. The surface of silica was most frequently functionalized with various organosilanes containing functional groups with high affinity to gold, such as amino (-NH_2_) or mercapto (-SH) group [11,17], or using polymers containing multiple -NH_2_ groups, such as polyethyleneimine (PEI) [18] or poly(diallyldimethylammonium chloride) (PDADMAC) [19]. In most cases, the organosilanes-containing amino group were used for functionalization because they provided uniform and relatively high surface coverage (up to 30%) with gold nanoparticles, which is very important for obtaining smooth continuous shell.

Most of the studies regarding a two-step process focused on the influence of the gold salts reducing agents on the shell formation. In the first studies on synthesis of silica-gold core-shell nanostructures, sodium borohydride was used as a reducing agent [11], however one of the most often used reducing agent in SiO_2_@Au nanostructures synthesis is formaldehyde [20,21,22]. Due to the toxicity of formaldehyde, researchers have also looked for safer alternatives to it, such as glucose [23] or ascorbic acid [24]. Also, hydroxylamine [25] and CO [26] were used as reducing agents. Use of gaseous CO as reducing agent results in uniform shell formation [26].

Only a few studies regarding direct growth of gold shell on the non-functionalized silica surface have been reported. These studies include the reduction of gold (I) chloride in acetonitrile with ascorbic acid [27], formaldehyde stimulated shell growth on Sn-seeded silica particles [28] and ultrasound assisted deposition of gold nanoparticles on silica particles surface [29]. Similarly, studies on direct growth of gold shell on amino-functionalized silica particle are limited to a few articles. For example, gold salts were directly reduced on functionalized surface of SiO_2_ particles using formaldehyde [30]. An interesting approach involves direct growth of gold shell on functionalized silica surface by reduction of gold salts with trisodium citrate [31,32,33]. Trisodium citrate was used by Turkevich [34] and Frens [35] for the synthesis of monodispersed gold nanoparticles and has become probably one of the most often used reducing agents for this purpose. It is not surprising since it is cheap, not hazardous, by varying its concentration one can control the size of synthesized gold nanoparticles, and, most importantly, it acts as both reducing agent and stabilizing agent. In studies reported by Zhang *et al.* [31], gold salts were reduced directly on the surface of amino-functionalized silica particles (average diameter of 227 nm) and the silica particles decorated with gold particles (average diameter of 14 nm) at 100 °C by rapidly injected trisodium citrate. In both cases rather non-uniform coverage with gold was achieved, however the coverage was higher in case of reduction on silica particles decorated with gold nanoparticles. Storti *et al.* [32] synthesized SiO_2_@Au core-shell nanoparticles by gold salts reduction on amino-functionalized silica nanoparticles (average diameter 16 nm) obtained in a one-step process. The formation of complete gold shell in these studies was carried out by slow addition of HAuCl_4_ solution to silica particles suspension containing trisodium citrate at 100 °C [32]. In studies of Zhang *et al.* [33], gold shell was grown on the tris(2,2′-bipyridyl)ruthenium(II) chloride embedded amino-functionalized SiO_2_ particles (diameter ~70nm). In this method, reducing agent was added dropwise to mixture containing functionalized cores and gold salt. Au shells of different thickness were obtained by adjusting the ratio of the SiO_2_ NPs and gold salt, while maintaining trisodium citrate in excess.

Herein, we report studies on the application of modified Turkevich synthesis method of Au nanoparticles to direct fabrication of SiO_2_@Au core-shell nanostructures. The effect of reaction temperature and amount of silica particles used in the reaction on deposition of gold nanostructures on the amino-functionalized silica particle was investigated. In addition, this method was for the first time applied to synthesis of TiO_2_@Au core-shell nanostructures.

## 2. Results and Discussion

**Figure 1:**
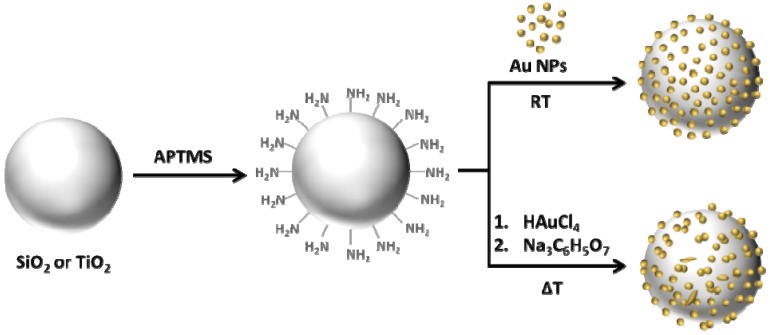
Schematic illustration of SiO_2_@Au and TiO_2_@Au core-shell nanostructures fabrication via decoration of NH_2_-functionalized cores with AuNPs and via direct reduction of gold salts.

The synthesis of SiO_2_@Au and TiO_2_@Au core-shell nanostructures described in this article was carried out according to procedures shown in Figure 1. The commercially available submicrometer silica and titania (rutile) particles, which are used as cores, were functionalized with -NH_2_ groups. The amino groups served as anchor groups, which bind gold nanoparticles to surface of functionalized SiO_2_ and TiO_2_ particles. In the first method used in these studies, amino-functionalized cores were decorated with 16–20 nm AuNPs synthesized using Turkevich method [34]. These particles served as reference structures to nanostructures obtained by direct reduction of gold salts on functionalized core using modification of Turkevich method. The syntheses of SiO_2_@Au and TiO_2_@Au nanostructures via direct reduction were carried out at different temperatures. In addition, the influence of different amounts of silica cores on the morphology of gold shell was investigated.

### 2.1. Deposition of AuNPs on Nh_2_-Functionalized Silicon Dioxide and Titanium Dioxide Cores

**Figure 2:**
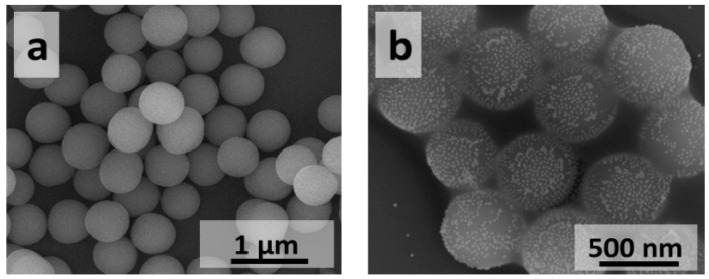

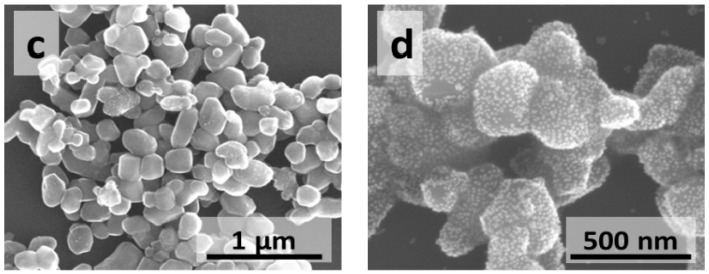
Scanning Electron Microscopy (SEM) images of silicon dioxide and titanium dioxide cores before and after deposition of Turkevich AuNPs: (**a**,**b**) SiO_2_ and (**c**,**d**) TiO_2_.

The silicon dioxide and titanium dioxide cores shown in Figure 2a,c were functionalized with amino groups and mixed with Turkevich AuNPs to yield structures shown in Figure 2b,d. For both core materials, the deposition yields structures with monodispersed gold nanoparticles evenly distributed on the surface of SiO_2_ and TiO_2_ cores. Gold nanoparticles are usually well separated on the SiO_2_ core surface, however in some areas of core surface clusters containing from two up to several AuNPs are also observed. This phenomenon is more pronounced in the case of TiO_2_ cores, where AuNPs are more densely packed. The degree of surface coverage with AuNPs and the way AuNPs are distributed on the core surface are dependent on the surface modifier used for core surface functionalization [36] and can be controlled by amount of AuNPs used for this process.

Different gold coverage on the SiO_2_ and TiO_2_ cores may be associated with the different content of OH groups on the surface of non-functionalized SiO_2_ and TiO_2_ cores, which may be up to four times higher for TiO_2_ [37,38,39]. The higher number of OH groups on the surface of TiO_2_ particles may result in higher number of NH_2_ groups after functionalization step and thus provide more anchoring groups for AuNPs binding.

**Figure 3:**
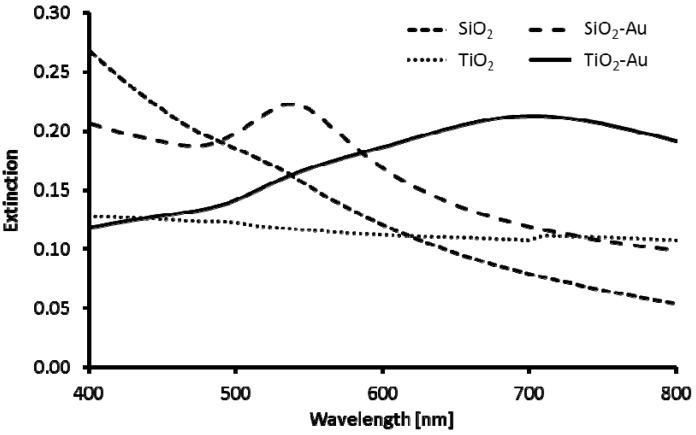
UV-Vis spectra of silicon dioxide and titanium dioxide cores before and after deposition of Turkevich AuNPs.

The UV-Vis spectra of silicon dioxide and titanium dioxide cores before and after deposition of Turkevich AuNPs are shown in Figure 3. Due to separation of AuNPs on the surface of silica cores, UV-Vis spectrum of SiO_2_@Au nanostructures closely resembles spectrum of AuNPs (not presented) and has extinction band centered at about 550 nm. The significant red shift of the maximum of absorption (λ_max_ = 670 nm) was observed in case of TiO_2_@Au nanostructures. The spectral location of plasmon resonance of single gold nanoparticle is dependent on the refractive index of surrounding medium. Therefore, the red shift observed for TiO_2_@Au in the UV-Vis spectrum as compared to SiO_2_@Au is likely related to the higher refractive index of TiO_2_ (n ~ 2.2–2.6) particles compared to SiO_2_ particles (n ~ 1.45). In addition, both red shift and broadening of the spectrum may be associated with dense packing of AuNPs on TiO_2_ particles surface resulting in Au nanoparticles plasmon-plasmon coupling.

### 2.2. Direct Reduction of Gold Salts on NH_2_-Functionalized Silicon Dioxide Cores

**Figure 4:**
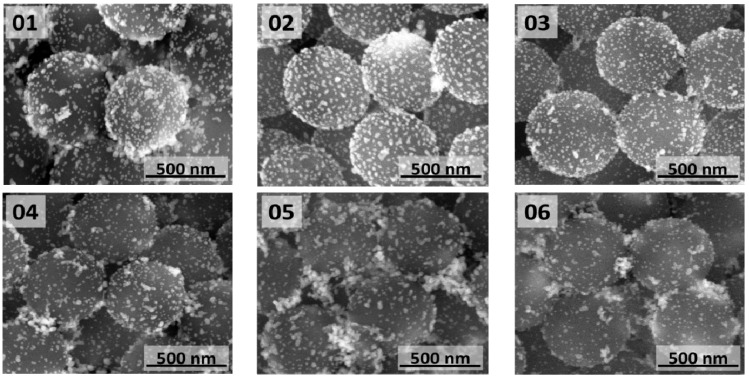
SEM images of SiO_2_@Au nanostructures obtained by direct reduction of gold salts on functionalized SiO_2_ particles at various temperatures: (**01**) 100 °C; (**02**) 90 °C; (**03**) 80 °C; (**04**) 70 °C; (**05**) 60 °C; and (**06**) 50 °C.

**Figure 5:**
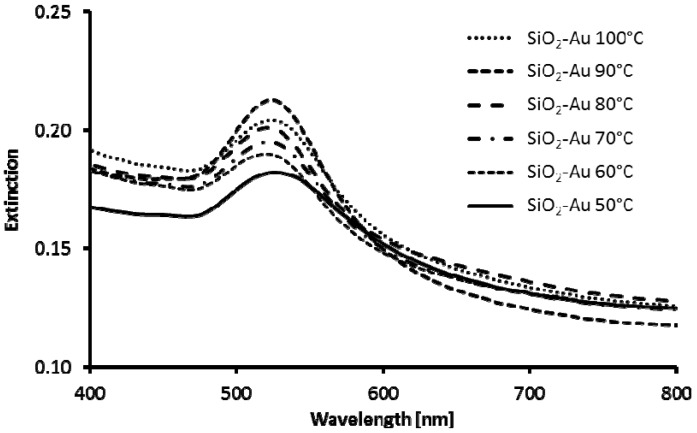
UV-Vis spectra of SiO_2_@Au nanostructures obtained by direct reduction of gold salts on functionalized SiO_2_ particles at various temperatures: (**01**) 100 °C; (**02**) 90 °C; (**03**) 80 °C; (**04**) 70 °C; (**05**) 60 °C; and (**06**) 50 °C.

The SiO_2_@Au nanostructures obtained by direct reduction of gold salts on functionalized SiO_2_ particles at temperatures in the 50–100 °C range are shown in Figure 4. For all temperatures, in addition to desired core-shell structures, formation of large amount of gold nanoparticles not attached to SiO_2_ particles was observed. The aim of lowering the reaction temperature was to slow down gold salts reduction process and therefore to provide more time for gold seeds to bind to NH_2_-functionalized silicon dioxide cores. As a result, higher coverage of cores with gold was expected. However, the opposite effect was observed. Denser and more uniform coverage was observed for higher reaction temperatures 80–100 °C. In these temperatures, syntheses were more reproducible. Direct reduction of gold salts on SiO_2_ cores at higher temperatures yields SiO_2_@Au nanostructures with denser coverage of gold as compared to SiO_2_@Au nanostructures obtained by AuNPs deposition. In addition, the gold nanostructures fabricated on core surface by direct reduction are of different shape and sizes, ranging from a few to several nanometers. Despite the fact that variety of gold nanostructures were obtained on SiO_2_ cores, their UV-Vis spectra are very similar to the UV-Vis spectrum of silica cores decorated with AuNPs (Figure 3 and Figure 5).

**Figure 6:**
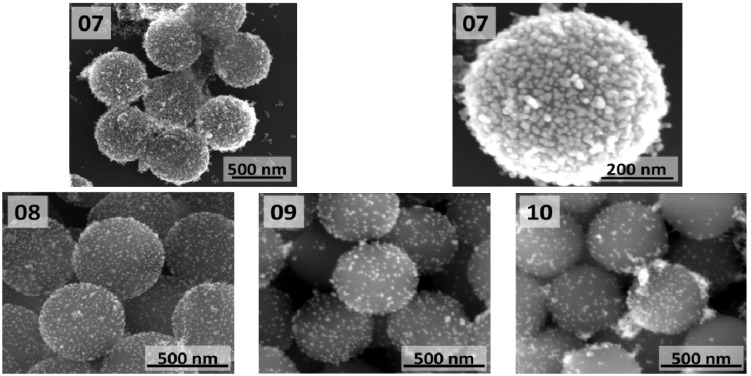
SEM images of SiO_2_@Au nanostructures obtained by direct reduction of gold salts in reaction mixture containing different amounts of functionalized SiO_2_ particles: (**07**) 2.5 µg/mL; (**08**) 13 µg/mL; (**09**) 50 µg/mL; and (**10**) 125 µg/mL.

The SiO_2_@Au nanostructures obtained by direct reduction of gold salts at 90 °C and 100 °C in reaction mixture containing different amounts of functionalized SiO_2_ particles (2.5, 13, 50 and 125 µg per mL of reaction mixture) are shown in Figure 6. These core-shell nanostructures could also be compared to SiO_2_@Au nanostructure shown in Figure 4 (02). The highest coverage of core surface was obtained when only 2.5 µg/mL of silica core particles were used (Figure 6 (07)). In these conditions, semicontinuous gold film formed on the silica surface. Unfortunately, due to low number of used silica cores, it was impossible to measure UV-Vis spectra of these structures. Structures with such shell morphology are expected to have high extinction in broad spectral range extending to longer wavelengths [40,41]. Based on SEM images (Figure 6), it is clearly seen that by increasing number of functionalized cores, lower gold coverage is achieved and also less Au particles and larger separation between them are observed. The UV-Vis spectra of (08)–(10) are qualitatively the same as for the (03)–(04) samples, but with less pronounced AuNPs extinction peak.

The comparison of the results obtained in these studies, syntheses (1)–(10), to previously reported studies [31,32,33] on SiO_2_@Au fabrication using trisodium citrate as reducing agent of gold salts yields some interesting observations. The method described by Zhang *et al.* [31], which closely resembles method reported in this article, yields SiO_2_@Au nanostructure with rather low surface coverage with gold, even in case of deposition of AuNPs on functionalized cores. In the studies reported here, surface coverage seems to be higher. These differences could be associated with the methods used for the functionalization of silica core. In our studies, functionalization of core surfaces in toluene allowed obtaining higher coverage with gold nanoparticles than in the case of functionalization in ethanol used in Zhang studies. It is quite difficult to relate the here reported studies to earlier studies [31,32,33] where trisodium citrate was used as a reducing agent. For all three cases, cores of different sizes, different silica surface functionalization methods, and reaction conditions were used.

### 2.3. Direct Reduction of Gold Salts on NH_2_-Functionalized Titanium Dioxide Cores

**Figure 7:**
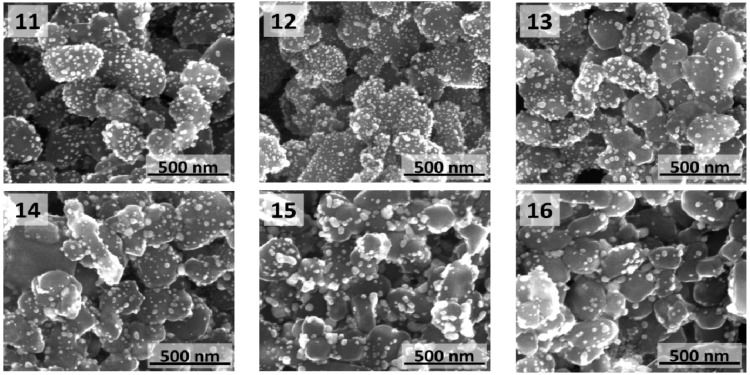
SEM images of TiO_2_@Au nanostructures obtained by direct reduction of gold salts on functionalized TiO_2_ particles at various temperatures: (**11**) 100 °C; (**12**) 90 °C; (**13**) 80 °C; (**14**) 70 °C; (**15**) 60 °C; and (**16**) 50 °C.

The TiO_2_@Au nanostructures obtained by direct reduction of gold salts on functionalized TiO_2_ particles using trisodium citrate at temperatures in the 50–100 °C range are presented in Figure 7. To the best of our knowledge, it is the first time this method is used for synthesis of TiO_2_@Au nanostructures. In our studies on the synthesis of TiO_2_@Au core-shell structures, we have applied many methods that were used for SiO_2_@Au [16]. However, in many cases methods, which worked fine for SiO_2_ failed for TiO_2_. It is related to the fact that opposite to chemically inert SiO_2_, TiO_2_ exhibits photocatalytic properties even in amorphous form. Because of that, formaldehyde, most commonly used reducing agent in case of SiO_2_, did not work in our experiments for TiO_2_. From the other hand, as shown in studies reported here, use of trisodium citrate yields TiO_2_@Au core-shell structures with relatively high gold coverage.

Similarly to studies for silica cores, the aim of lowering temperature was to slow down gold salts reduction process and therefore provide more time for gold seeds to bind to NH_2_-functionalized titanium dioxide cores. As a result, higher coverage of cores with gold was expected. However, it was observed that by lowering reaction temperature, less gold nanostructures were formed on the surface of TiO_2_ cores and, in addition, the size of AuNPs increased as the temperature was lowered. Lower number of gold nanostructures attached to cores observed for lower temperature reactions may be related to unbinding of large gold nanostructures from the core surface during the work up of a reaction. It seems that denser surface coverage with gold was observed when AuNPs were deposited on the NH_2_-functionalized titanium dioxide cores (Figure 2d).

**Figure 8:**
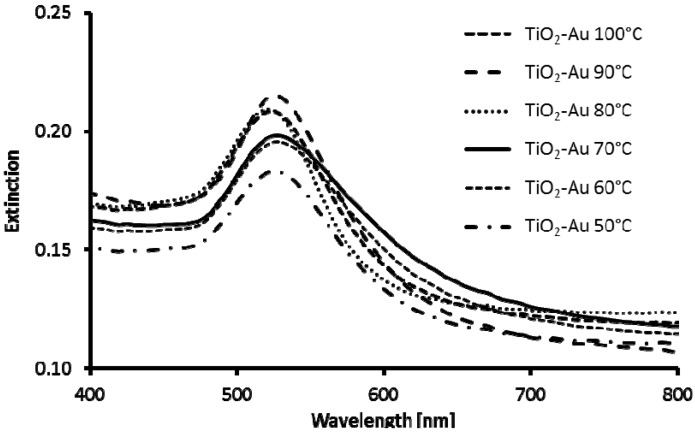
UV-Vis spectra of TiO_2_@Au nanostructures obtained by direct reduction of gold salts on functionalized TiO_2_ particles at various temperatures: (**11**) 100 °C; (**12**) 90 °C; (**13**) 80 °C; (**14**) 70 °C; (**15**) 60 °C; and (**16**) 50 °C.

The UV-Vis spectra of TiO_2_@Au nanostructures obtained by direct reduction (10–15) (Figure 8) correspond well to spectrum of AuNPs and differ significantly from spectra of TiO_2_ cores decorated with AuNPs (Figure 3). In all cases, small red shifts of absorption maximum (λ_max_) as compared to spectrum of AuNPs are observed, which is likely caused by larger size of gold nanostructures on the TiO_2_ surface compared to Turkevich AuNPs and TiO_2_ refractive index being higher than that of water.

## 3. Experimental Section

### 3.1. Reagents

Silica particles (spherical particles with average diameter of 670 nm), titania particles (rutile particles of irregular shape and size in range of 100–200 nm) and trisodium citrate were purchased from POCH S.A. (3-Aminopropyl)-trimethoxysilane (APTMS) was purchased from Sigma Aldrich. Hydrogen tetrachloroaurate(III) trihydrate (HAuCl_4_) was purchased from Alfa Aesar and was used to prepare 5% dark-aged stock solution of chloroauric acid, later used in the experiments. Other reagents were of analytical grade. All the chemicals were used without further purification. Deionized (DI) water (resistance > 18.2 MΩ) used in all synthesis and washing was prepared by an ultrapure water system HLP 5UV (Hydrolab).

### 3.2. Functionalization of Silica and Titania Submicroparticles

Silica and titania submicroparticles were functionalized with amine groups using modified method described by Jaroniec *et al.* [17]. The silica or titania particles (0.5 g) were added to 50 mL of anhydrous toluene in Falcon tube, which was then placed in ultrasonic bath and sonicated for 1 h. The suspension was transferred to round bottom flask and allowed to stir vigorously for 1 h. Next, the 2.5 mL of APTMS was added to mixing suspension. Such reaction mixture was boiled under reflux for 24 h. After completion of the reaction, modified particles were washed several times on a membrane filter with small portions of toluene and ethanol to remove an excessive amount of modifier. The functionalized particles were dried overnight in an oven at ~100 °C.

### 3.3. Deposition of Turkevich AuNPs on Silica and Titania Particles

Gold nanoparticles were synthesized according to Turkevich method [34]. Briefly, 1 mL of solution of 5 mM HAuCl_4_ in DI water was added to 18 mL of DI water and mixture was heated until it began to boil. One milliliter of 0.5% trisodium citrate solution was added as soon as boiling commenced. The reaction mixture was heated until evident color change had occurred and then it was removed from heating plate and stirred until it cooled down to room temperature.

Five milligrams of amine-functionalized silica or titania particles were dispersed in 10 mL of DI water under ultrasound for 1 h. Next, 4.5 mL of the silica or titania functionalized particles suspension was added to 30 mL of Turkevich AuNPs solution and mixed for a few hours. In order to remove AuNPs unattached to silica or titania particles surface, the reaction mixture was subjected to a few washing steps by centrifugation and redispersion in 0.5% solution of trisodium citrate.

### 3.4. Direct Deposition of Au Nanostructures on Silica and Titania Particles Using Turkevich Method

In typical synthesis, 1 mL of 5 mM HAuCl_4_ aqueous solution was added to 17 mL of DI water and mixed for 5 min before addition of 1 mL of water suspension containing 0.5 mg of amine-functionalized silica or titania particles. Resulting reaction mixture was heated until it reached the selected reaction temperature in the 50–100 °C range and then the preheated 1 mL of 0.5% trisodium citrate solution was added. The reaction mixture was heated until evident color change had occurred and then it was removed from heating plate and continued to stir until it cooled down to room temperature. In order to remove AuNPs unattached to silica or titania particles surface the reaction mixture was subjected to a few washing steps by centrifugation and redispersion in 0.5% solution of trisodium citrate. Conditions of (01)–(16) syntheses described in article are given in Table 1 and Table 2.

**Table 1:** Reaction temperatures and amount of SiO_2_-NH_2_ particles used in the syntheses 01–10.

Sample Reaction Conditions	01	02	03	04	05	06	07	08	09	10
SiO_2_-NH_2_ (µg/mL)	25	25	25	25	25	25	2.5	13	50	125
T (°C)	100	90	80	70	60	50	100	90	90	90

**Table 2:** Reaction temperatures and amount of TiO_2_-NH_2_ particles used in the syntheses 11–16.

Sample Reaction Conditions	11	12	13	14	15	16
TiO_2_-NH_2_ (µg/mL)	25	25	25	25	25	25
T (°C)	100	90	80	70	60	50

### 3.5. Characterization

The UV–Vis extinction spectra were measured at room temperature using Lambda 900 UV-Vis-NIR spectrophotometer (Perkin Elmer, Waltham, MA, USA) in the 400–800 nm spectral range. Suspensions of synthesized nanostructures were measured in 1 cm optical path quartz cuvette. The morphology of SiO_2_-Au and TiO_2_-Au nanostructures was characterized based on the images obtained using a Quanta 3D FEG Dual Beam scanning electron microscope (FEI, Hillsboro, OR, USA). Samples for SEM were prepared by drop-casting of suspensions of core-shell nanostructures on silicon wafer and drying in air.

## 4. Conclusions

In this paper, we applied Turkevich method for AuNPs synthesis to fabrication of SiO_2_@Au and TiO_2_@Au nanostructures. The nanostructures synthesized using this method were compared to SiO_2_@Au and TiO_2_@Au nanostructures obtained by deposition of Turkevich AuNPs on amino-functionalized SiO_2_ and TiO_2_ cores. The nanostructures fabricated using both methods have different morphologies of gold shell and exhibit different optical properties. In the case of SiO_2_ cores, both methods may yield core-shell structures with similar optical properties. However, by control of reaction conditions, it is possible to obtain core-shells with semicontinuous gold shell, which are expected to have high extinction in broad spectral range. Interestingly, differences in optical properties were observed for TiO_2_@Au structures. The significant red shift and broadening of the spectrum in the case of AuNPs decorated TiO_2_ cores are likely related to high refractive index of TiO_2_ and dense packing of AuNPs on TiO_2_ particles surface, resulting in strong AuNPs plasmon-plasmon coupling. Modified Turkevich method yields core-shell nanostructures with dense surface and high reproducibility at temperatures in the 80–100 °C range. To the best of our knowledge, this is the first report describing the use of Turkevich method to direct fabrication of TiO_2_@Au core-shell nanostructures.
